# Unstained Blood Smear Analysis: A Review of Rule‐Based, Machine Learning, and Deep Learning Techniques

**DOI:** 10.1002/jbio.202500121

**Published:** 2025-06-30

**Authors:** Husnu Baris Baydargil, Thomas Bocklitz

**Affiliations:** ^1^ Institute of Physical Chemistry (IPC) and Abbe Center of Photonics (ACP), Friedrich Schiller University Jena Germany; ^2^ Leibniz Institute of Photonic Technology, Member of Leibniz Health Technologies, Member of the Leibniz Centre for Photonics in Infection Research (LPI) Jena Germany

**Keywords:** biophotonics, deep learning, machine learning, rule‐based image analysis, unstained blood smear analysis

## Abstract

Blood cells are central to oxygen transport, immune defense, and hemostasis. Their number and morphology act as sensitive biomarkers, making accurate segmentation and classification essential for hematological diagnostics. Biophotonic techniques now provide label‐free imaging of unstained smears by exploiting intrinsic phase and scattering contrast, yet such images exhibit low optical signal and subtle morphological variation that exacerbate segmentation errors. Label‐free modalities nevertheless preserve contrast where dyes fail, motivating renewed interest in unstained workflows. This review analyzes rule‐based, machine‐learning, and deep‐learning approaches for segmenting and classifying label‐free blood cells, highlighting performance gains, persistent challenges, and future directions for clinical adoption.

## Graphical abstract

Bright‐field images of unstained smears. (1) Sparse erythrocytes allow straightforward intensity‐ or phase‐based segmentation. (2) Overlapping cells blur boundaries, causing over‐ or under‐segmentation and lowering rule‐based accuracy, thus motivating overlap‐aware algorithms for reliable downstream feature extraction and classification.

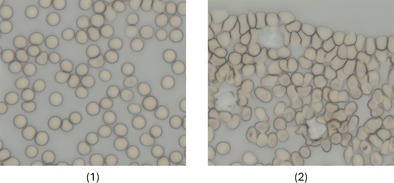

## 1 Introduction

Blood plays a fundamental role in maintaining physiological balance by transporting oxygen, nutrients, hormones, and waste products throughout the body. It comprises various components, including red blood cells (RBCs), white blood cells (WBCs), platelets, and plasma, each serving distinct functions critical for homeostasis. Quantitative and qualitative evaluations of blood components provide essential diagnostic insights into a wide range of medical conditions, from infections and anemia to hematological malignancies [1]. Because blood smears are inexpensive and widely available, light‐microscopic examination of a thin film remains one of the first‐line tests in clinical laboratories [2].

Traditionally, blood‐smear evaluation depends on cytochemical stains, most commonly with Giemsa and Wright–Giemsa dyes which bind nucleic acids, parasite pigment, and cytoplasmic granules, creating high‐contrast colors that let microscopists differentiate leukocyte lineages, grade anemia, and identify malaria down to < 50 parasites μL^−1^ [3]. Standardized stain recipes and reading criteria have been embedded for decades in external‐quality‐assessment and accreditation documents, most notably CLSI guideline H56‐A, which mandates pH‐controlled buffers, reference control slides, and inter‐laboratory concordance testing to ensure reproducible results across sites [4]. Stained smears are therefore deeply integrated into routine hematology: they are inexpensive, teachable with minimal instrumentation, and compatible with existing automated differentials that flag abnormal films for manual review [5]. Their drawbacks are equally well recognized. Preparation adds roughly 10–15 min and has reagent costs; methanol fixation irreversibly alters cell morphology and precludes longitudinal or drug‐response studies; and repeated staining can vary in quality, particularly when laboratories accelerate protocols to meet throughput targets [6]. These practical limitations motivate the exploration of label‐free, biophotonic alternatives that aim to match stained‐smear diagnostic performance while eliminating time, reagent, and fixation barriers.

Against this backdrop, a growing body of biophotonic techniques, defined as the generation, manipulation, and detection of light to study biological specimens, addresses these limitations by exploiting the intrinsic response of unstained cells. Quantitative phase imaging (QPI) [7] and digital holographic microscopy (DHM) [8] render nanometer‐scale height or refractive‐index variations, while optical diffraction tomography (ODT) [9] reconstructs three‐dimensional refractive‐index maps. Bright‐field microscopy remains popular for its simplicity but offers lower intrinsic contrast than phase‐based methods [10]. Therefore, advanced segmentation and classification algorithms are critical for extracting meaningful diagnostic information from label‐free images.

Extending the discussion beyond purely phase‐ or scatter‐based contrast, the diagnostic value of label‐free imaging is highly modality‐dependent. Vibrational techniques such as Raman spectroscopy and coherent anti‐Stokes Raman scattering (CARS) contribute molecular‐specific signals, most notably from lipids and proteins that are invisible to quantitative‐phase or bright‐field microscopy [11, 12]. Recent multimodal studies have demonstrated that acquiring a CARS channel on the same smear already imaged by QPI or bright‐field microscopy enables joint algorithms to fuse morphology with chemistry, thereby boosting cell‐type separation and parasite detection without dyes [13, 14]. Because a CARS acquisition adds only a few seconds and requires no reagents, laboratories can deploy it as a secondary, label‐free quality control layer: slides are first screened digitally in the unstained state, and only those yielding ambiguous predictions advance to full Giemsa or Wright staining for confirmatory review. This dual‐pathway workflow shortens turnaround, conserves staining chemicals, and provides an internal safeguard against fixation artifacts, underscoring how, in the near term, modality‐tailored, unstained imaging will complement rather than replace the stained gold standard in routine clinical hematology.

Label‐free approaches complement rather than replace the stained gold standard. They eliminate reagents, preserve live‐cell physiology for kinetic or drug‐response assays, and mesh easily with high‐throughput microfluidic or imaging‐flow‐cytometry platforms that can screen tens of thousands of cells per second [15, 16]. However, to gain broad clinical acceptance, they must match stained‐smear accuracy and reproducibility and integrate into existing accreditation pathways.

Leveraging these richer signals, researchers have redesigned segmentation and classification pipelines to be modality aware. For instance, retraining a support‐vector machine on QPI‐derived optical‐path‐length histograms raised malaria‐infected RBC detection in bright‐field images from 88% to 95% [8]. In DHM, Sobel‐guided phase‐gradient maps combined with Dice‐optimized watershed achieved a Dice score of 0.95 for rat RBC segmentation in micro‐flow, outperforming intensity‐only rules by more than 10%. Volumetric ODT tomograms unlock 3D convolutional networks that classify five WBC subtypes with 96.7% accuracy in < 0.2 s per cell [17].

Beyond the laboratory, photonics‐AI integration is moving towards devices usable in the field. The handheld Remoscope cytometer combines a compact QPI module with an embedded neural network to screen two million unstained erythrocytes for Plasmodium falciparum in under 12 min [18], while phase‐aware digital‐staining networks such as *PhaseStain* convert QPI maps into Giemsa‐like color images that pathologists can interpret without additional reagents [19]. Together, these examples show that modern unstained blood analysis is a biophotonics with AI progress in which optical‐contrast engineering and algorithmic advances co‐evolve to deliver clinically relevant performance.

Segmentation and classification are fundamental processes in automated blood cell analysis. Segmentation separates individual cells or sub‐cellular regions, which enable quantitative extraction of size, shape, texture, and in‐phase modalities, and dry mass [20]. Accurate segmentation ensures that subsequent classification tasks, where cells are categorized based on their type or pathological state are, reliable. Classification then assigns each object to a biological category, from malaria life stage to leukemic blast, to provide clinically actionable counts [21]. Continuous advancements in segmentation and classification methodologies make it possible for efficient, reliable, and scalable hematological diagnostic workflows in clinical and research settings.

Rule‐based methods represent the earliest approaches for automated blood cell analysis, relying on predefined algorithms that utilize image processing techniques to detect and segment cellular structures. These methods often employ thresholding, edge detection, and morphological operations to differentiate cells based on shape, size, and texture. Techniques such as Otsu's thresholding [22] and the watershed algorithm [23] have been widely used due to their simplicity and computational efficiency [24, 25, 26]. However, while effective for images with clear boundaries and uniform contrast, rule‐based methods struggle with complex scenarios involving overlapping cells, variable lighting conditions, and heterogeneous cell morphologies, limiting their accuracy in diverse clinical samples [13, 27]. These limitations highlight the need for more adaptive approaches that could handle greater variability and complexity in blood smear images such as ML.

ML techniques introduced a paradigm shift by enabling data‐driven feature extraction for classification and segmentation. Early ML models, such as support vector machines (SVMs) [14] and Naive Bayes classifiers [28], and k‐nearest neighbors (k‐NN), relied on handcrafted features like cell shape, texture, and intensity for classification and segmentation tasks [29, 30, 31]. These models demonstrated improved performance in handling overlapping cells and varying morphologies compared to traditional methods [32]. However, their dependency on feature engineering and limited ability to generalize across different datasets pose challenges, especially when dealing with large‐scale or highly heterogeneous samples [33]. To overcome these challenges, DL evolved as a specialized subset of ML by offering end‐to‐end learning capabilities and improved adaptability to large and complex datasets.

DL has revolutionized blood cell analysis by enabling automatic feature extraction and end‐to‐end learning from raw image data. Convolutional neural networks (CNNs) [34], in particular, have shown exceptional performance in segmentation and classification tasks due to their ability to capture hierarchical features such as edges, textures, and complex morphological patterns. Architectures such as U‐Net [35] have been widely adopted for cell segmentation, offering robust performance even in challenging conditions such as variable contrast and cell overlapping [36, 37]. Unlike traditional ML models, DL approaches do not rely on manual feature engineering, allowing them to generalize better across diverse datasets. However, they require large amounts of annotated data for training and are computationally intensive, which can limit their accessibility in resource‐constrained settings. Additionally, the “black box” nature of DL models poses challenges in interpretability and transparency, although ongoing research into explainable artificial intelligence (XAI) aims to provide deeper insights into model decision‐making processes.

The aim of this article is to trace how segmentation and classification strategies have co‐evolved with advances in label‐free biophotonics, and to identify the remaining bottlenecks that prevent routine clinical deployment. It critically examines the strengths and limitations of each method in addressing the unique challenges posed by unstained imaging, including low contrast and subtle morphological variations. Furthermore, this review explores the integration of various imaging modalities, algorithmic advancements, and data augmentation strategies to improve diagnostic accuracy. By identifying current gaps and emerging trends, this work seeks to inform the development of more robust, efficient, and clinically applicable solutions for automated blood cell analysis for improved diagnostic workflows and patient outcomes in hematological analysis.

## 2 Survey Methodology

To conduct a comprehensive and systematic review of blood cell segmentation and classification methodologies, the following approach was adopted:

**Sources:** Literature search was performed using reputable databases, including PubMed, Google Scholar, IEEE Xplore, and ScienceDirect to ensure broad and credible coverage.

**Keywords:** The search strategy incorporated combinations of keywords such as “unstained” “label‐free” “segmentation” “classification” “machine learning” “deep learning,” and to capture a wide range of relevant techniques and innovations.

**Search Refinement:** Keywords were strategically combined into multiple queries, with iterative refinement to maximize the inclusion of relevant studies across diverse methodologies and applications.

**Selection Criteria:** Studies were selected based on their relevance, contribution to the field, and scientific rigor. Priority was given to recent publications that demonstrate improved performance, address key challenges, or introduce state‐of‐the‐art techniques.

**Bias Mitigation:** To ensure an unbiased representation of the literature, efforts were made to include diverse methodologies and perspectives.

**FIGURE 1:**
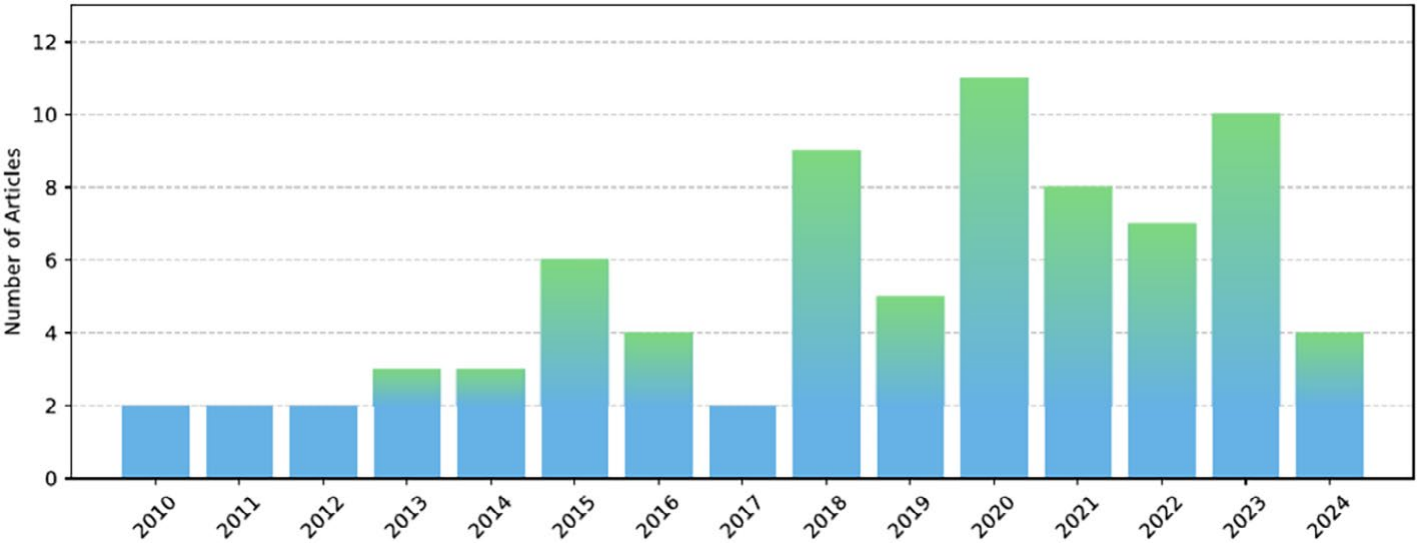
The annual distribution of published articles related to unstained blood smear analysis.

**Finalization:** A curated list of articles was compiled for detailed analysis with a mix of rule‐based, machine learning, and deep learning methodologies. A total of 78 relevant articles were initially identified across multiple sources, and a refined selection of 45 was made to maintain methodological rigor and diversity. Specifically, PubMed yielded 36 articles, from which 12 were selected; Google Scholar identified 41 articles, with 14 selected; IEEE Xplore contributed 11, with 7 selected; and Scopus provided 25, with 12 selected. This final selection forms the foundation of the review, encompassing advancements in blood cell segmentation, classification, and detection. The yearly distribution of articles can be seen in Figure 1.

## 3 Evaluation Metrics and Benchmarking

Automated analysis of unstained blood smears covers three distinct computer‐vision subtasks: segmentation, detection, and classification. For pixel‐wise segmentation, region‐overlap indices such as the Dice coefficient and Intersection‐over‐Union (IoU) are the prevailing standards. They capture how much of a cell mask is correctly recovered [38]. Object‐detection studies then wrap IoU into the mean Average Precision (mAP), which allows performance to be averaged across size scales. Classification pipelines use class‐specific precision and recall, which are usually condensed into macro or weighted‐F1 scores. These rely on a single “accuracy” figure that can gloss over clinically dangerous errors; an algorithm that misses every ring‐stage malaria parasite may still score 99% accuracy because healthy erythrocytes dominate the smear [39].

Overlap scores can hide boundary slips that affect morphometrics. Boundary‐aware measures such as the 95th‐percentile Hausdorff distance [40] penalize the worst contour deviations and are informative when cells overlap or when dry‐mass calculations rely on precise perimeters [41]. Therefore, reporting both region and boundary‐based metrics gives a better picture of segmentation quality.

Class imbalance amplifies the need for thoughtful averaging schemes. In Ryu et al.'s work [42], leukocytes make up less than 0.2% of 115,k labelled cells, while in Doan et al.'s [43] storage‐lesion study smooth discs outnumber crenated forms five to one. Under such skew, a single “accuracy” figure can hide clinically dangerous errors; an algorithm that misses every ring‐stage malaria parasite may still score 99% accuracy because healthy erythrocytes dominate the smear. Metrics that average per‐class performance, macro‐ or weighted‐F1, balanced accuracy or the precision‐recall area under the receiver operating characteristic curve (AUROC), are therefore recommended by recent guidelines on medical‐image evaluation [44]. Moreover, when possible, supplementation of calibration curves, Expected Calibration Error (ECE) or Brier score may be vital because they highlight whether probability outputs can be trusted in workflows [45].

Robust assessment also demands data that the model has not seen during training. A systematic review of external‐validation studies found a consistent 3–7 percentage‐point drop in F1 when algorithms were applied to donor‐held‐out or multi‐center datasets, showing the gap between laboratory accuracy and clinical robustness [46]. Therefore, including an external slide set, or at minimum a patient‐level hold‐out split, should become standard practice, particularly for label‐free modalities where acquisition settings vary widely between microscopes.

Finally, metric reporting must be tied to optical context. Bright‐field RGB, QPI phase maps, DHM gradient images, and ODT refractive‐index volumes inhabit different statistical spaces; a single ImageNet‐style benchmark is unlikely to emerge soon. Instead, modality‐specific, versioned micro‐benchmarks released with preprocessing code and metric scripts will allow fair, future‐proof comparison as biophotonic hardware evolves and ensure that improvements in Dice score, mAP, or macro‐F1 translate into safer, faster hematology.

## 4 Rule‐Based Methods

Rule‐based methods have been foundational in the development of automated blood cell segmentation and classification systems. These methods rely on predefined heuristics derived from expert knowledge of cellular morphology, color, and texture [47]. The deterministic nature of rule‐based algorithms allows for interpretable decision‐making, making them especially useful in controlled environments where imaging conditions are consistent. Despite their simplicity, low computational cost, and ease of implementation, rule‐based approaches often face limitations when dealing with noisy images, overlapping cells, and variations in imaging conditions [48, 49]. Moreover, they lack generalizability, as new imaging conditions may require the development of additional rules, limiting their adaptability to diverse datasets. To address the inherent limitations of rule‐based methods, researchers have developed tailored segmentation techniques that enhance accuracy in specific scenarios.

Historically, rule‐based methods addressed the limitations of manual microscopy by offering faster processing and reducing inter‐observer variability by applying a sequence of logical conditions, such as intensity thresholds, geometric constraints, or color space transformations, to identify regions of interest within microscopic images [50].

Building on classical distance‐transform watershed, Lina et al. [51] first normalized color variation by converting RGB images to HSV, then sharpened faint cytoplasmic edges with Laplacian filtering before imposing a nucleus‐centered marker map. This multi‐step pre‐conditioning was crucial: the raw watershed routinely merged neighboring cells, whereas the marker‐guided version preserved individual leukocytes even in sparse clusters. Nevertheless, the authors note that indistinct nuclear membranes in lymphocytes often led the algorithm to split a single cell into fragments, illuminating an intrinsic weakness of intensity‐only cues in label‐free smears.

**FIGURE 2:**
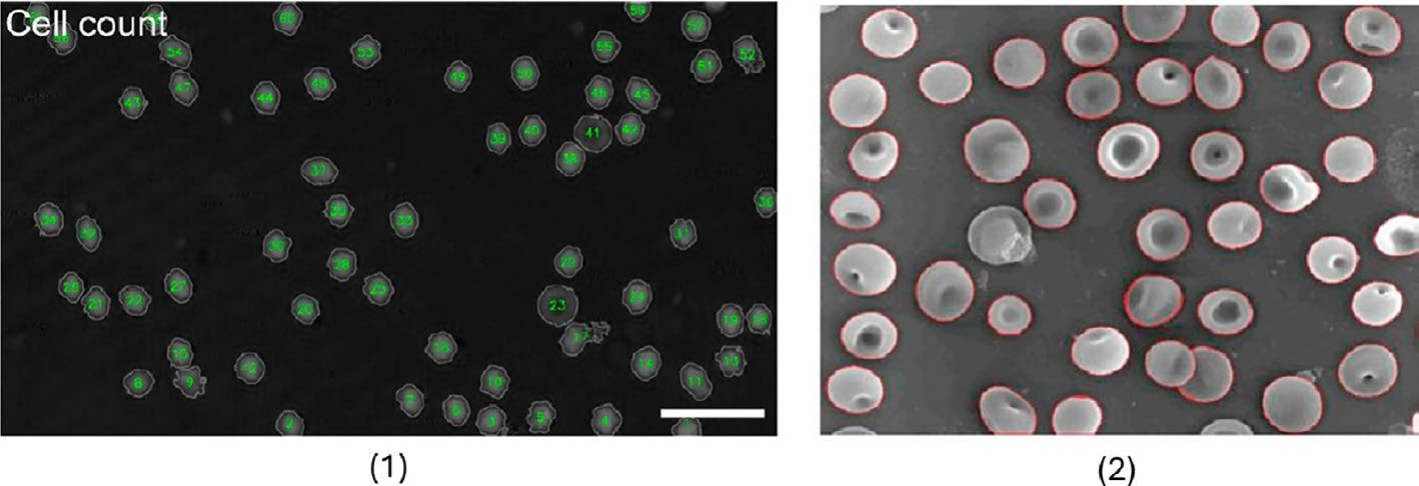
Representative rule based benchmarks on unstained blood smears. (1) Automated erythrocyte counting and contour extraction in quantitative phase digital holographic microscopy images, obtained with an adaptive Otsu threshold followed by a distance transform watershed by Xiong et al. [50], and RBC subtype segmentation in scanning electron micrographs using a marker controlled watershed pipeline by, Bhowmick et al. [52]. Image 1 acquired from “Automatic identification and analysis of cells using digital holographic microscopy and Sobel segmentation” by Xiong Z. et al. Frontiers in Photonics, 5, 1 359 595 (2024), CC BY 4.0. Image 2 reproduced from Bhowmick S. et al. Micron 44, 384–394 (2013). Elsevier, reproduced with permission.

In a SEM‐based variant of watershed, Bhowmick et al. [52], derived 47 morphological and textural descriptors, among them entropy of grey‐level co‐occurrence and 3D shape factors that fed a Bayesian classifier for anemia sub‐typing. Their marker‐controlled watershed used valleys in the gradient magnitude to seed segmentation, which worked well on crenated or sickled erythrocytes but less so on normocytic‐normochromic cells, due to smooth perimeters lacking the surface indentations needed for marker placement. The reliance on SEM hardware and manual cell pick‐up underscores why this pipeline has yet to see clinical uptake despite its high analytical precision. Typical outputs from Xiong et al. and Bhowmick et al.'s work are displayed in Figure 2.

Additionally, Doumun et al. [53] exploited the Beer–Lambert attenuation model across 13 illumination wavelengths to flatten spectral inhomogeneities, thereby creating a “pseudo‐phase” map that accentuated erythrocyte rims. A two‐stage threshold (local Otsu followed by hysteresis contour closure) produced crisp binary masks that the authors fed into a size‐adaptive watershed. This spectral normalization makes the pipeline robust to lamp flicker and slide‐to‐slide variation, but it also amplifies small gradients, so heavily overlapped cell groups were occasionally split into three or more fragments. The authors suggest adding curvature‐based concavity pruning in future iterations to suppress this over‐segmentation.

**FIGURE 3:**
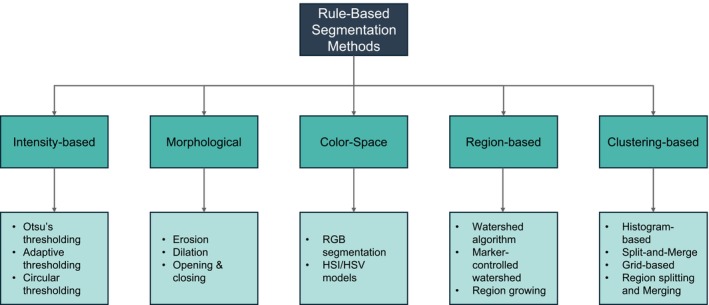
A diagram illustrating rule‐based segmentation methods commonly applied in unstained blood smear analysis. These techniques provide a structured approach to identifying cellular structures. Rule‐based methods are particularly useful in unstained samples where contrast is inherently low, as they can enhance feature visibility through mathematical transformations and region‐growing techniques. While effective in controlled conditions, these approaches often struggle with overlapping cells, uneven illumination, and subtle morphological differences, They remain valuable as interpretable and computationally efficient benchmarks for evaluating more advanced ML and DL‐based approaches.

One of the most commonly used rule‐based techniques is intensity‐based segmentation, which distinguishes cellular structures from the background by analyzing pixel intensity distributions. Thresholding is a primary approach in this category, where pixels are classified based on their intensity values relative to a predefined threshold. Otsu's global method determines the optimal threshold by maximizing between‐class variance [24]. While effective for images with uniform contrast, it struggles with overlapping cells and uneven illumination. Adaptive thresholding techniques improve segmentation performance in heterogeneous samples but face challenges with dense overlaps. A schematic overview of the rule‐based strategies discussed in this section is provided in Figure 3.

Moon et al. [49] leveraged off‐axis digital holographic microscopy to trace storage‐induced lesions in red blood cells without any staining. Eleven blood‐bag cohorts (8–57 days, > 300 cells each) yielded 3300 quantitative‐phase volumes (60 ×/0.85 NA, 682 nm), from which individual RBCs were isolated by an adaptive Otsu threshold, distance‐map watershed, and concavity pruning. Automated extraction of 3D morphometric and biochemical indices, projected area, optical‐volume‐derived MCV, mean corpuscular hemoglobin (MCH) and its surface density revealed that shape and MCH surface density change sharply after 34 days, whereas bulk hemoglobin content remains stable. The study shows how a label‐free, rule‐based workflow can generate thousands of per‐cell measurements that inform blood‐bank shelf‐life; however, it reports no formal segmentation accuracy metric and depends on stable off‐axis coherence, which limits portability to other DHM setups.

Yi et al. [47] demonstrated that conventional distance‐transform watershed can be highly effective when paired with the intrinsic contrast of off‐axis DHM. Working with 38 quantitative‐phase maps, each a 512 × 512 pixel reconstruction containing 180 unstained erythrocytes, the authors applied adaptive Otsu thresholding to the phase image, seeded a distance‐map watershed from local minima, and pruned false fragments by concavity analysis, achieving 96.4% segmentation accuracy and 93% cell‐detection efficiency relative to manual masks. Because the output includes per‐cell phase profiles, the same pipeline enables dry‐mass estimation without additional staining or chemical markers. Performance nevertheless depended on stable coherence length and focus: when the off‐axis angle or illumination bandwidth was altered, threshold levels and watershed markers required re‐tuning, and densely packed clusters occasionally split into fragments. The work highlights both the promise and the portability limits of rule‐based segmentation in truly label‐free, phase‐rich environments (Table 1).

**TABLE 1:** Sample rule‐based methods for segmentation and classification in unstained blood smear analysis. This table compares selected rule‐based segmentation algorithms, highlighting their performance metrics, key strengths, and challenges. Rule‐based methods are included here due to their foundational role in image analysis, which provides baseline performance benchmarks against more advanced methodologies, such as ML and DL. Despite their limitations with complex or noisy data, they remain valuable for understanding morphological characteristics and informing improvements in automated diagnostic workflows.

Article	Methods	Performance	Strengths	Challenges
Lina et al. [51]	Modified watershed segmentation Image enhancement (HSV transformation, morphological operations) **Dataset (bright field)** 458 unstained 722 stained	Proposed algorithm: **65.72%** unstained **94.82%** stained CNN model: **65.33%** unstained **74.85%** stained	Combined image enhancement improves performance Comparative performance to CNNs	Difficulty in detecting lymphocytes (17.65% acc) CNN showed lack of generalizability (testing accuracy on unseen images 56%)
Moon et al. [49]	Off‐axis DHM phase imaging (60×/0.85 NA, 682 nm) Adaptive Otsu threshold Distance‐map marker‐controlled watershed Concavity pruning Extraction of 3D morphometrics (area, volume) and biochemical indices (MCH, MCH surface density) over 11 storage‐age classes Dataset Over 3300 RBCs Classes range from 8 to 57 days	No explicit segmentation accuracy reported Statistical analysis over 3300 RBCs shows significant morphologic change beyond 34 days of storage	Purely label‐free, non‐invasive Large, balanced dataset (> 300 cells per age class) Generates simultaneous morphology and hemoglobin metrics useful for blood‐bank QC	Rule thresholds must be returned for different DHM optical paths No quantitative segmentation benchmark or external validation Mid‐age classes overlap, limiting discriminative power
Doumun et al. [53]	Beer–Lambert's law with statistical standardization Local adaptive thresholding Hysteresis contour closing Watershed algorithm for segmentation **Dataset (multispectral, unstained transmission images)** 30 images from 13 LEDs (7×1020×768×23×2048×1088)	Accuracy: **96.75%** Precision: **98.47%** Recall: **98.23%** F‐1 Score: **98.34%**	Does not require extensive preprocessing Effective in handling overlapping cells Processing time of under 2 min for images with size 2048 × 1088	Over‐segmentation in high gradient areas Potential sensitivity to imaging mode Performance variability with image size
Bhowmick et al. [52]	Marker‐controlled watershed algorithm for segmentation of erythrocytes 47 feature extractions including 24 textural, 17 geometric, and 5 entropy‐based **Dataset (SEM bright field)** 200 images (5 anemia classes, private)	Accuracy: **88.99%** Sensitivity: **94.95%** Specificity: **100%** Positive Predictive Value (PPV): **100%** Segmentation Efficiency: **92%–95%**	High precision in subgroup classification Combination of multiple types of features Effective in handling overlapping cells	Reliance on pathologists for pre‐selecting anemic cells Overlapping cells require extensive preprocessing Lower accuracy for normocytic normochromic anemia Requires SEM hardware/prep
Yi et al. [47]	Off‐axis DHM phase imaging Background subtraction Adaptive otsu + distance‐map watershed Contour‐pruning **Dataset: (38 full‐field DHM frames)** 512 × 512 px 1180 micrographs at 1000× oil magnification (pixel size = 0.25 μm; raw frame 2592 × 1944 px) 458 images left unstained; 722 Wright‐Giemsa stained	Segmentation accuracy = **96.4%** Detection efficiency = **93%**	Purely label‐free Handles dense microflow Quantitative phase enables downstream dry‐mass estimates	Rule parameters sensitive to coherence/focus Occasional over‐splits in tight clusters Single‐lab dataset, no external validation

Edge detection algorithms such as the Canny edge detector [54], Sobel operator [55], and Laplacian of Gaussian [56] are widely used in medical imaging, although they are not as common in unstained blood smear analysis.

Xiong et al. [50] demonstrated a purely Sobel‐based pipeline on QPI‐DHM images: a Dice‐optimized Sobel threshold segmented 43 711 flowing, unstained rat erythrocytes at roughly 0.05 s per 96 × 96 frame, achieving an F1 of 0.95 and a Hausdorff distance of 6.4 px versus manual masks while simultaneously yielding dry‐mass and concentration read‐outs. Because the method relies only on intrinsic optical‐path differences, it avoids dye artefacts and shows that physics‐driven edges can still compete when paired with simple optimization. However, the method is sensitive to precise focus; performance falls more than 10% Dice if the hologram is reconstructed ±1 μm from the optimal plane and has limited robustness to dense clumps (> 4 cells), which occasionally merge despite threshold tuning. This indicates that additional concavity pruning or soft‐clustering would be needed for clinical‐grade throughput.

Beyond algorithm design, open‐access data availability remains a barrier. Anzaku et al. [57] addressed this by publishing the open‐access Tryp corpus, 3085 positive and 93 negative thick‐smear images of Trypanosoma brucei, each accompanied by bounding‐box annotations and released under a permissive CC‐BY license. Captured on two markedly different microscope rigs (an Olympus IX83 research system and a CKX53 fitted with a smartphone adapter), the archive intentionally preserves real‐world variability in resolution, field‐of‐view, and illumination. Using Tryp, the authors benchmarked YOLOv7, YOLOv5, and Faster R‐CNN, with YOLOv7 reaching an mAP of 0.92 yet still failing on motion‐blurred parasites and under‐exposed frames. The dataset therefore serves a dual purpose: it supplies the community with hard‐to‐obtain label‐free images and exposes the kinds of artefacts that future rule‐based or ML pipelines must withstand.

Open repositories such as Tryp are critical for reproducibility and fair benchmarking, but they also raise practical questions about patient privacy, informed consent, and uniform annotation standards. While blood‐smear images rarely contain directly identifiable information, ethical data‐sharing agreements are still needed to cover sample provenance and potential pathogen risks. Moreover, Tryp currently provides bounding boxes rather than pixel‐accurate masks, meaning researchers interested in instance‐level segmentation must either crowd‐source new annotations or develop weakly supervised approaches. Filling these annotation and governance gaps will be just as important as algorithmic innovation if label‐free blood‐smear analysis is to progress from proof‐of‐concept to a clinically robust tool.

Handling overlaps remains a crucial rule‐based problem. Marker‐controlled watershed, concavity analysis, and related heuristics have been deployed to split clumped cells [58, 59]. For instance, Yeubet et al. [60] approached the issue optically: their spectral time‐multiplexed super‐resolution (TMSR) bright‐field holography illuminated unstained Plasmodium smears with eight laser wavelengths, reconstructing 0.5 μm‐resolution phase maps whose SNR improved by 7 dB over single‐wavelength images. The sharper phase gradients let a Canny detector raise mean Dice overlap from 0.78 to 0.92 on 150 test images, while also revealing hemozoin for stage identification. The trade‐off is complexity; synchronized lasers and eight‐frame capture limit portability and throughput. However, the study shows how physics‐driven contrast can enhance classical edges.

**FIGURE 4:**
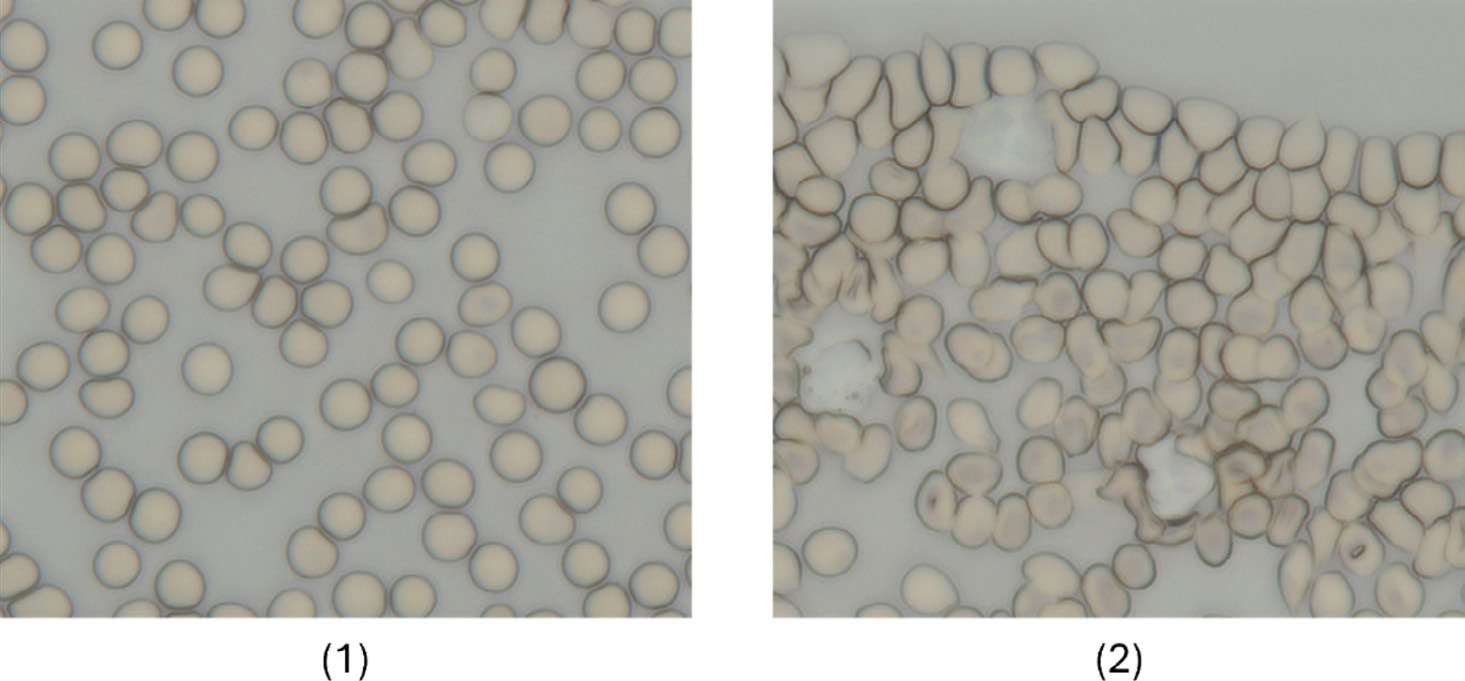
Representative unstained thin‑film blood smears acquired with bright‑field microscopy. (1) A sparse field in which individual erythrocytes are well separated. Such images allow straightforward intensity or phase‑based segmentation and reliable measurements (diameter, projected area, optical path length). (2) A clumped field where cells overlap or touch, producing blurred or merged boundaries and shadowing artefacts. Overlaps reduce local contrast, confound edge detectors and watershed markers, and can lead to over‑segmentation (one cell split into fragments) or under‑segmentation (multiple cells merged), ultimately pushing errors into feature extraction and machine or deep‑learning classifiers. The contrast difference between (1) and (2) illustrates why most rule‑based pipelines report markedly lower accuracy on clinical slides, where clustering is common, and motivates the development of overlap‑aware algorithms.

Even with modern developments, rule‐based pipelines demand precise, modality‐specific tuning, and tend to fail when confronted with new imaging artifacts. The practical impact of these challenges is visible in Figure 4, where a sparse field allows easy threshold‐based segmentation, whereas clumped erythrocytes blur boundaries and confound even modern DL detectors. Machine‐learning (ML) methods sidestep handcrafted rules by learning feature representations from data, and deep‐learning (DL) architectures push this further with end‐to‐end optimization, capabilities we examine in the next section.

## 5 | Machine Learning Methods

**FIGURE 5:**
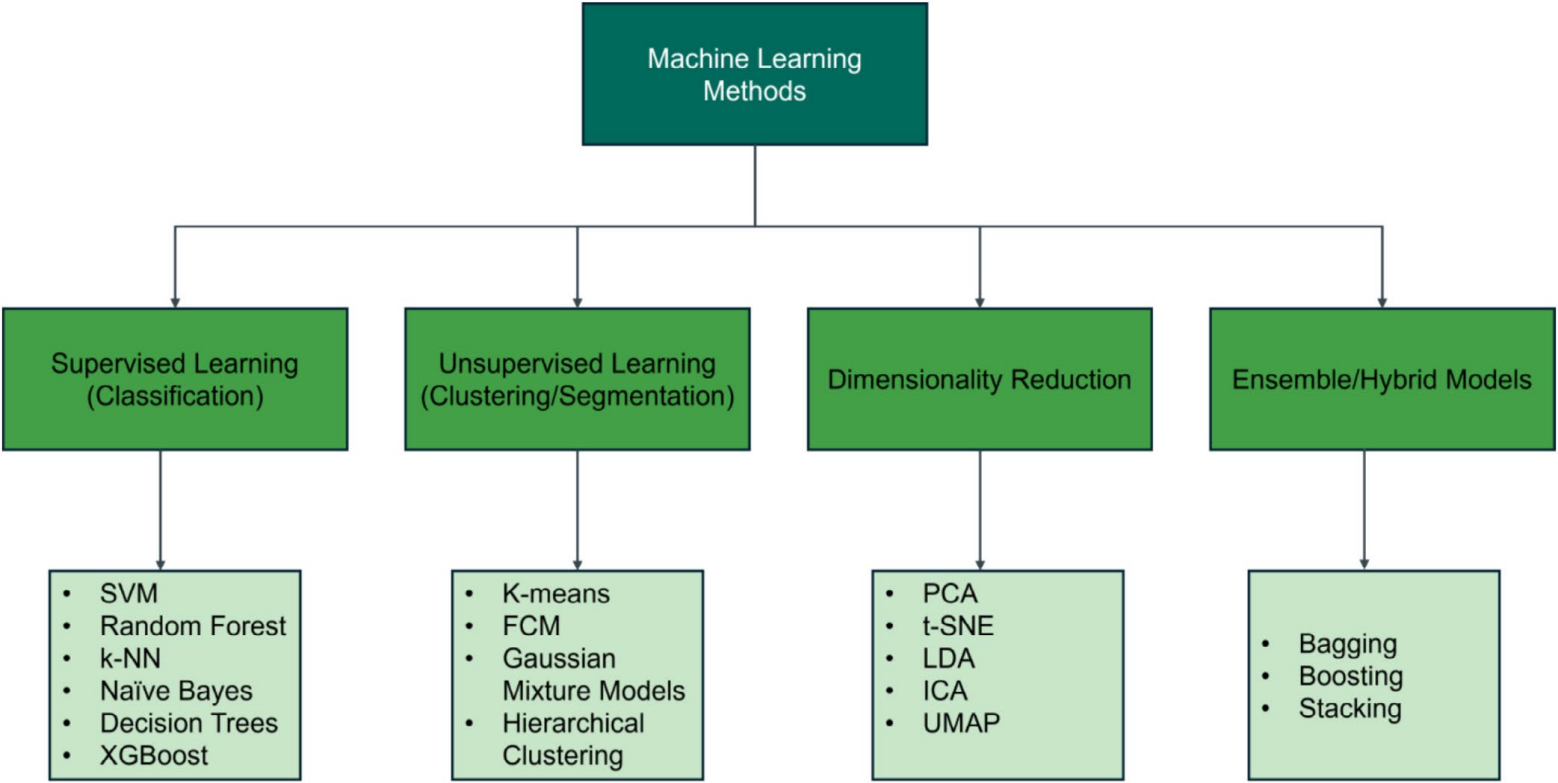
A diagram illustrating prominent ML methods categorized into classification, clustering/segmentation, dimensionality reduction, and ensemble models. Supervised learning techniques classify blood cells based on morphological and textural features, useful in automated diagnostics. Unsupervised learning methods segment unstained blood smears by grouping similar cells without labeled data, making them essential for exploratory analysis. Dimensionality reduction techniques preserve critical features while minimizing noise and computational complexity, improving efficiency for large‐scale imaging datasets. Ensemble and hybrid models enhance robustness and generalizability by combining multiple algorithms.

Machine learning (ML) has significantly advanced the analysis of blood cells by introducing robust frameworks for segmentation and classification tasks. While segmentation focuses on accurately delineating cell boundaries and structures within microscopic images, classification aims to categorize these segmented regions based on morphological or biochemical features. Traditional ML approaches, such as SVM and kNN, have demonstrated effectiveness in classifying WBCs based on morphological features like shape, size, and texture. These models rely heavily on handcrafted feature extraction, where domain expertise is essential for designing features tailored to specific diagnostic objectives. Accurate segmentation not only improves the precision of classification models but also ensures that machine learning techniques receive high‐quality inputs, ultimately improving diagnostic accuracy. Figure 5 highlights the most popular ML methods for classification, clustering, dimensional reduction, and ensemble of multiple & hybrid models.

Traditional ML methods for blood cell classification often rely on robust feature extraction techniques. Zheng et al. [21] demonstrated this on a 43‐image set of unstained bright‐field smears (2048 × 1536 px, ~80–100 RBCs per frame). Their two‐stage scheme first localized cells with a Viola–Jones+AdaBoost histogram of oriented gradients (HOG) [61] cascade, reaching 93% detection accuracy and processing one frame in ≈3 seconds; a second stage used HOG morphology features to label each cell infected or healthy, yielding infection sensitivities between 75% and 82%. The approach coped well with varying parasite stages but stumbled on low‐contrast, irregularly shaped uninfected cells, and the small, single‐laboratory dataset limited generalizability and produced unbalanced sensitivity versus specificity. The study underscores that even when feature engineering boosts classifier accuracy, accurate upstream segmentation and larger, more diverse training sets remain essential for robust label‐free malaria screening.

Lippeveld et al. [15] employed an ImageStream‐XMKII imaging‐flow cytometer (IFC) to acquire stain‐free bright and dark‐field images at a throughput of 5000 cells per second and compiled two benchmark datasets. The first dataset, WBC‐8, contains 95 000 focused single leukocytes drawn from eight immunophenotyped classes: 59 000 neutrophils, 17 000 CD4^+^ T cells, 8000 CD8^+^ T cells, 4300 B cells, 2700 monocytes, 2200 CD56^+^ NKT cells, 1300 other NKT cells, and 3200 eosinophils. The EOS set comprises 190 000 events that are heavily imbalanced toward non‐eosinophils (186 000) with 1291 active and 2595 resting eosinophils. From each image, the authors extracted 213 *CellProfiler* [62] features and compared traditional ensemble classifiers with deep neural networks: on five‐fold cross‐validation, gradient‐boosting and random‐forest models achieved balanced accuracies of 77.8% and 77.4%, respectively, on WBC‐8 and outperformed ResNet‐18 (77.4%) and the specialized DeepFlow network (85.6% on EOS but 77.4% on WBC‐8). A UMAP projection of the handcrafted feature space showed substantial intra‐class heterogeneity, explaining why both ML and DL models struggled to separate closely related subtypes. The study shows a high‐throughput, label‐free acquisition pipeline, open data release, and the insight that classic ensemble methods can rival or exceed CNNs when datasets are modest or imbalanced. Limitations include the relatively coarse 90 × 90 pixel crops, the dependence on fluorescence labels for ground truth, and the limited generalizability caused by strong class imbalance.

**FIGURE 6:**
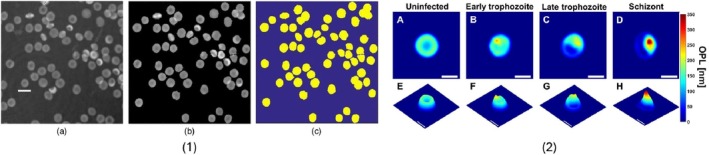
Examples of two ML‐based methods for unstained blood smear analysis: (1) segmentation of quantitative phase images (QPI) of red blood cells (RBCs) obtained by off‐axis digital holographic microscopy (DHM) using a marker‐controlled watershed algorithm, as demonstrated by Ahmadzadeh et al. [64] for morphology‐based clustering; and (2) detection of malaria parasite stages and visualization with QPI by Park et al. [8]. Image (a) reproduced from Ahmadzadeh et al. [64], J. Biomed. Opt. 22(7), 076015 (2017), DOI: 10.1117/1.JBO.22.7.076015, licensed under CC BY 4.0. Image (b) from Park et al. [8], 2016, licensed under CC BY).

Ozaki et al. [63] used reflection‐type quantitative phase microscopy (QPM) to image 250 healthy donor WBCs and 250 cells from five cancer lines (SW480, DLD‐1, HCT116, Panc‐1, HepG2) at 0.27 μm pixel sampling. After triangle‐threshold segmentation, each single‐cell phase map was longitudinally normalized (OPL/PL and OPL/D), resized to 49 × 49 px, and described either by five statistical moments or by a 1296‐dimensional HOG feature vector. A linear‐kernel SVM trained on HOG of OPL/PL achieved 96.8% accuracy (AUC = 0.996), outperforming statistical‐feature models (94.2%) and a Random‐Forest baseline. The pipeline showed impressive sub‐cellular morphometry and label‐free acquisition, but performance was constrained by the small sample (*n* = 500), susceptibility to slight defocus, and absence of overlapping‐cell cases, which may suggest the need for larger and more heterogeneous QPM datasets before clinical deployment. Representative results from Ahmadzadeh et al.'s [64] and Park et al. [8] pipelines are highlighted in Figure 6.

Extending Raman‐based ML beyond morphology, Happillon et al. [65] analyzed unstained May‐Grünwald–Giemsa blood smears with confocal Raman micro spectroscopy at 532 nm to flag chronic lymphocytic leukemia (CLL) directly on slides. From 49 CLL patients (4257 lymphocyte spectra) and 27 healthy volunteers (2628 spectra) they background‐subtracted, smoothed, and SNV‐normalized each spectrum, then used a random features search to retain the most discriminative wavenumbers (DNA and protein bands). A Gaussian‐kernel ν‐SVM trained on the reduced feature set yielded 90.9% sensitivity and 84.6% specificity on a validation set, and 80% sensitivity/100% specificity on an independent prediction set, while a companion model distinguished lymphocyte from polymorphonuclears with over 98% accuracy. This work shows truly label‐free acquisition, molecular interpretability (higher nucleic‐acid peaks in CLL nuclei), and evaluation on separate prediction smears. On the other hand, a drop in sensitivity is observed for borderline cases, and the pipeline relies on single‐point nuclear spectra (no spatial context), and the need for high signal‐to‐noise 532 nm measurements limits throughput.

Segmentation serves as a foundational step in the analysis pipeline, as accurate delineation of cellular boundaries directly impacts the performance of subsequent classification tasks. K‐means clustering is a popular unsupervised technique that partitions the image into k number of clusters by minimizing intra‐cluster variance [66, 67, 68]. In a label‐free setting, Ahmadzadeh et al. [64] recorded off‐axis DHM phase maps of 275 unstained erythrocytes and extracted five morphology descriptors, average cell thickness, projected surface area, mean corpuscular volume, sphericity coefficient, and perimeter, and then reduced them to three principal components. Applying K‐means clustering on this reduced (Table 2).

**TABLE 2:** Sample ML methods for segmentation and classification in unstained blood smear analysis. These methods illustrate the diversity in segmentation, classification, and preprocessing strategies, highlighting the necessity of manual feature extraction in traditional ML approaches. ML techniques mitigate some limitations of rule‐based methods through adaptive, data‐driven features, though they still depend heavily on domain expertise for designing effective features.

Article	Method	Performance	Strengths	Challenges
Zheng et al. [21]	Stage 1: HOG for feature extraction, AdaBoost for classification of malaria‐infected RBCs Stage 2: Comparative analysis of multiple feature descriptors and classification approaches **Dataset (unstained bright‐field micrographs)** 43 images (24 training, 19 testing) with resolution 2048×1536	Cell Detection: Cascade object detector: **93%** Hough Transform**: 69%** Infected Cell Classification (sensitivity): Viola‐Jones + Hough Transform + Intensity Thresholding: **82.14%** Viola‐Jones + Hough Transform + Red Channel Thresholding: **75%** Viola‐Jones Only: **42.86%**	Comparative approach enables the analysis of multiple methods Adapts well to different stages of malaria infection Average processing time of 3.3 ± 0.4 s/image	Over‐reliance on feature descriptors Small dataset Unbalanced sensitivity and specificity No pixel‐level masks; dot‐labels only, limiting supervised segmentation training
Park et al. [8]	Off‐axis Mach‐Zehnder QP spectroscopy (QPS) to capture morphological features of malaria‐infected RBCs Three classification models performance comparison (LDC, LR, kNN) **Dataset** $\left(107\times 1024^{2},0.12\;\mathrm{\mu m}\;\mathrm{px}\right)$: 1237 single RBCs from one donor culture (413 uninfected, 173 early, 314 late trophozoite, and 337 schizont)	Accuracy: Schizont Stage Detection: **99.7%** Late Trophozoites: **99.5%** Early Trophozoites: **98%** Specificity: **99.8%** for infection stage discrimination Sensitivity: **45.0%–66.8%** for early trophozoite stages	Deep characterization of cell morphology through 23 descriptors Analyze each cell in 15 s (benchmark model: 3000 s) Capable of distinguishing malaria stages	Single‐donor dataset may restrict generalization Limited early stage sensitivity (45% to 66.8%) Misclassification of WBCs as uninfected RBCs (33% to 89% rate) High sensitivity but limited generalization due to small sample size
Lippeveld et al. [15]	Using Amnis ImageStream‐X MK II IFC to capture 0.25 μm, 12 channels with a throughput of 5000 cells per second Comparative analysis of multiple ML and DL models Dimension reduction using UMAP **Dataset:** 8 class set (17–59 k cells per major subtype—strong eosinophil imbalance) bright & darkfield images	Balanced Accuracy: ML: Gradient Boosting: **77.8%** Random Forest: **77.4%** DL: ResNet18: **77.4%** DeepFlow: **85.6%**	UMAP highlights data heterogeneity Image preprocessing mitigates artifacts from brightfield channels The method of data handling allows batch training of models	DL models struggle to differentiate between similar cell subtypes such as CD4+ vs. CD8 + T‐cells Resolution choice of 90×90 might not capture fine cellular features Strong imbalance in EOS dataset (187 000 non‐EOS vs. 3900 EOS)
Nassar et al. [69]	Utilize IFC for WBC identification Extract 213 morphological features from shape, size, intensity, and texture using CellProfiler (ImageStream 100; 6 channels, focus metric threshold = 55, 0.3μmpx) Utilize multiple ML algorithms for classification **Dataset** Training‐13 donors (stained & unstained), Testing‐85 donors (unstained) with > 10 000 cells per donor	WBC classification: Gradient boosting: **97%** (F1 score) for main WBC types **78%** for subclassification of B and T cells)	Explainable feature selection analysis through CellProfiler Employment of random under sampling with majority voting Subject‐wise cross‐validation mitigates overfitting risks Dataset with over 1 million cell samples	Specific imaging and sampling protocols limits generalization High dependency on IFC Sensitivity to sample preparation variability such as imaging conditions, instrument calibration Performance sensitive to focus metric (≥ 55) and instrument calibration
Ozaki et al. [63]	Use Reflection‐type QPM (625 nm LED 0.27 μm px) to obtain morphological information of live, unstained cells Use triangle algorithm for initial cell segmentation SVM classifier with a linear kernel **Dataset** $\left(49\times 49\right)$: 250 WBCs (healthy donor) and 250 cancer cell images	Accuracy: Statistical OPL/PL: **94.2%** Statistical OPL/D: **87.2%** HOG OPL/PL: **96.8%** HOG OPL/D: **95.0%**	The use of HOG features improves the ability to capture subcellular heterogeneity Method differentiates WBCs from five cancer cell lines Explainable feature extraction method offers insights to cellular pathology	Results are highly preprocessing dependent Performance is negatively impacted by defocusing issues during QPM image acquisition Suboptimal performance with lower quality images Small sample size (*n* = 500)

Space grouped the cells into young (biconcave), stomatocyte, and senescent sphero‐echinocyte classes with 98.9% accuracy for two clusters and 95.2% for three clusters, matching manual labels without color stains. The pipeline handled moderate defocus and illumination drift thanks to scale‐invariant features and PCA normalization. Mis‐assignments occurred for transitional discocytes at the biconcave–stomatocyte boundary. The authors proposed coupling K‐means with fuzzy or density‐based methods to better capture these borderline morphologies.

Another label‐free study is the digital in‐line holographic microscopy (DIHM) work of Go et al. [7]. In this work, authors numerically reconstructed raw holograms of 200 healthy and 200 malaria‐infected RBCs; single cells were segmented by local peak searching and 13 morphological‐and‐scattering descriptors were extracted, 10 of which passed analysis of variance (ANOVA) feature selection. An SVM classifier trained on 280 cells and tested on 120 achieved 97.5% accuracy (96% correct segmentation, mean‐average precision (mAP) 0.975), outperforming five other traditional algorithms while requiring no staining or phase unwrapping. The DIHM setup is optically simple and records 50–100 cells per frame, giving higher throughput than interferometric QPI. Misclassifications were observed in early ring‐stage infections, and the eight‐step hologram reconstruction limits real‐time speed.

Fuzzy c‐means extends K‐means by assigning soft membership values to each pixel, a property that has been exploited in several stained‐smear studies. For instance, Mohapatra et al. [70] and Mondal et al. [71] have separated leukocyte sub‐regions with high accuracy in stained images. However, we found no primary paper applying FCM to truly label‐free or unstained blood‐smear images, leaving an obvious methodological gap for future work.

Despite these advancements, ML approaches face inherent limitations due to their dependence on handcrafted features. The manual design and selection of features are time‐intensive and may fail to capture the full variability of cell morphologies and imaging conditions. Moreover, while clustering and classification techniques have been successful in addressing specific challenges, their inability to handle clumped cells effectively underscores the need for more adaptive and automated methodologies. This limitation is further compounded by the scarcity of datasets with images of overlapped cells and corresponding ground truth masks, restricting the development of ML‐based methods for overlapping cell segmentation. To address these challenges, DL methods have been proposed that provide advanced solutions by automatically learning feature representations directly from raw image data, eliminating the need for manual feature engineering and demonstrating improved performance as more data becomes available, unlike traditional ML techniques.

## 6 Deep Learning Methods

Deep learning (DL) has emerged as a transformative technology in medical imaging, offering substantial advancements in the diagnosis and monitoring of hematological disorders. In blood image analysis, DL models facilitate tasks such as classification and segmentation of cellular structures, essential for identifying conditions like leukemia, anemia, and sickle cell disease [72, 73, 74]. These models can analyze intricate patterns in microscopic blood smear images, automating processes traditionally reliant on manual interpretation by experts. This automation not only improves diagnostic accuracy but also reduces the time required for analysis, paving the way for efficient and scalable healthcare solutions.

DL‐based image classification models aim to categorize images by learning representations that distinguish between different classes. Rather than directly extracting shape, texture, and structural morphology, CNNs such as ResNet [75] and EfficientNet [76] learn hierarchical feature representations through convolutional filters applied to input images. These filters capture local patterns like edges and textures, which are progressively combined through successive layers to form more complex and abstract representations. Pooling layers further reduce spatial dimensions while preserving critical local information, contributing to scale and translation invariance. The success of CNNs can be attributed to their image‐specific inductive bias, which improves their efficiency in managing scale invariance and capturing local visual features, even before training [77]. However, this localized focus limits their ability to model long‐range spatial dependencies effectively, which can negatively affect performance in tasks that require a broader contextual understanding [78]. Despite their strengths, CNNs are limited in modeling long‐range dependencies, affecting performance in tasks requiring broader contextual understanding. Additionally, CNNs require a large amount of data for effective training. While this is not a major issue for natural images, where labeled datasets are abundant, it becomes a significant challenge in medical imaging due to the limited availability of labeled data. This data scarcity makes it difficult to develop models that generalize well across different medical datasets [79].

**FIGURE 7:**
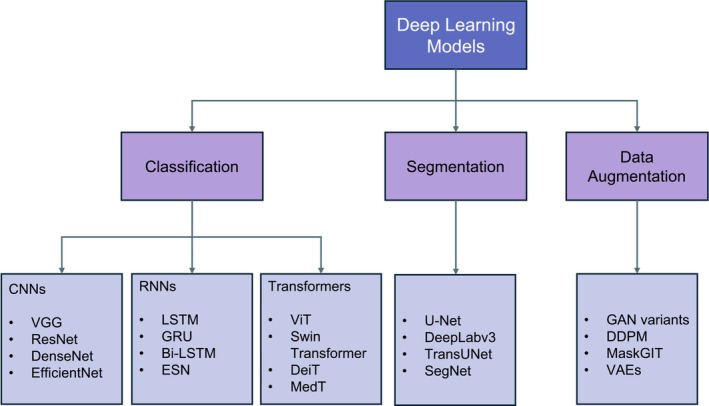
A diagram showing prominent DL models. In computer vision, classification models categorize blood cells based on morphological and textural features, typically using CNNs for spatial feature extraction and transformers for capturing long‐range dependencies and modeling global relationships between image regions. RNNs are applied in dynamic imaging tasks, such as time‐lapse microscopy or multi‐frame analysis, where sequential dependencies exist. Segmentation models perform pixel‐wise classification to delineate individual cells, effectively handling boundaries and overlapping structures. Generative models enhance data diversity and improve model generalizability by synthesizing realistic samples from the learned distribution, to address data scarcity issues.

**FIGURE 8:**
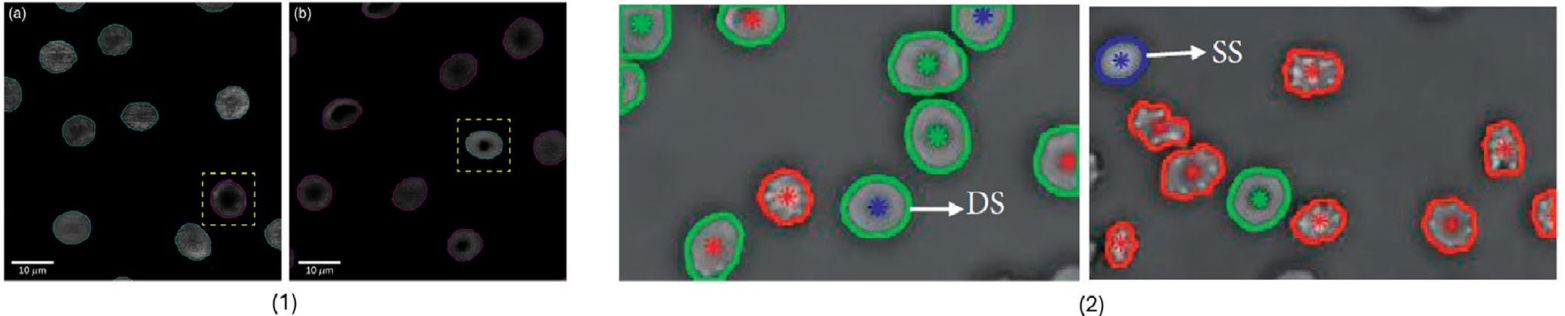
An example of two DL‐based benchmark articles: (1) Mask R‐CNN based detection and segmentation of thalassemic (tRBCs) versus healthy RBCs in QPI by Lin et al. [81], showing the model's delineation performance; and (2) automatic classification of RBC morphology (e.g., discocyte, echinocyte, spherocyte) from QPI using a stacked sparse autoencoder and boundary curvature analysis by Jiang et al. [82], with examples of classification outcomes. Image (1) reproduced from Lin et al. [81] (The Authors, 2020, published by SPIE, Journal of Biomedical Optics, DOI: 10.1117/1.JBO.25.11.116502, licensed under CC Attribution 4.0 Unported License.) Image (2) reproduced from Jiang et al. [82] 2022, International Journal of Optics, DOI: https://doi.org/10.1155/2022/1240020, licensed under CC BY 4.0.

While CNNs excel in feature extraction and classification, they can also be integrated into multimodal approaches to improve the system's ability to manage complex imaging structures. One notable approach to is the article by Ryu et al. [42], who developed a label‐free hematology pipeline that couples optical‐diffraction‐tomography (ODT) with the anchor‐free FCOS detector. Their dataset comprised 1224 three‐dimensional refractive‐index tomograms (0.162 μm lateral resolution) annotated with 115143 cells from four classes, RBC, abnormal RBC, platelet and WBC, split 70/20 / 10% for training, validation and test. With a ResNet‐18 backbone the model reached 0.970 mAP for detection and a weighted F1 = 0.971 (total accuracy = 0.971) for four‐class classification. A Swin‐Transformer [80] backbone improved mAP to 0.977 but increased parameters by ~65%. Morphological and biochemical indices derived from the RI maps correlated well with commercial analyzers (MCV *r* = 0.905, MCH *r* = 0.889), demonstrating quantitative validity. The pipeline includes true 3‐D input, has fast inference (~0.15 s per cell) and the first large‐scale ODT benchmark, however, the performance on the minority WBC class remains limited (124 instances) and the need for a specialized ODT rig restricts clinical portability. Moreover, ARBC labelling depends on subjective “irregular‐shape” criteria, showing the importance of clearer ground‐truth standards. Representative DL models for classification, segmentation, and data augmentation tasks can be seen in Figure 7. Qualitative outputs of Lin et al.'s [81] and Jiang et al.'s [82] work are highlighted in Figure 8.

Chen et al. [83] replaced the cumbersome dual‐beam optics of quantitative‐phase microscopes with a single, defocused bright‐field microscope that is illuminated by 415 nm light, the Soret absorption peak of hemoglobin. At a defocus of 1.1 μm, the microscope converts the second‐order derivative of the optical phase into intensity, yielding “blood defocusing phase‐contrast” (BDPC) images whose contrast is 2–3 times higher than that of conventional white‐light defocus. Using this low‐cost set‐up the authors recorded 3711 BDPC patches (512 × 512 px) from 35 unstained peripheral‐blood smears and trained a YOLOv5 object detection model to perform five‐part WBC differentiation. On a held‐out test set the network achieved mean F1 = 0.986 and mAP = 0.980, while automated counts on 14 additional smears correlated strongly with manual Wright–Giemsa tallies (*R* ≥ 0.90 for all classes except the sparsely represented basophils). Strengths include the use of a regular microscope, sub‐second exposure times, and the ability to visualize both leukocyte granules and hemoglobin distribution without stains; limitations are the need to fix the illumination at 415 nm, sensitivity to precise defocus (tolerance≈0.6 μm), and reduced performance on basophils because of extreme class imbalance.

Chen et al. [84] built a custom, label‐free bright‐field data set of single white‐blood‐cell images by first separating leukocytes in a spiral microfluidic chip, then acquiring paired fluorescence/bright‐field frames to derive ground‐truth labels for three classes, granulocytes, lymphocytes, and monocytes (≈2200 raw cells, expanded by rotation and flipping to 31 000 images after augmentation). Otsu thresholding cropped each cell to 200 × 200 px before training a feature‐fusion CNN that concatenates low‐level edge/textural maps from the early layers with high‐level semantic maps from deeper layers. On an 80/20 train–test split, the network reached 80.3% overall accuracy, recall ≈0.81, and macro‐F1≈0.80, outperforming three ablation variants and ResNet‐50 by 0.3–1.3 pp. The fused‐feature design preserved morphological detail; visualized activation maps show clearer nuclear contours than the deep baseline; however, the performance still lagged behind stained‐smear models (> 90%). Contributing factors were the modest class‐balanced sample size, reliance on fluorescence‐derived labels during data set creation, and limited augmentation (only flips/rotations); the authors suggest richer perturbations and, ultimately, 3D tomographic inputs to close the gap.

While 2D CNNs offer effectiveness and versatility, transitioning to 3D CNN architectures provides deeper insights by capturing volumetric data, an approach especially beneficial for analyzing complex cellular structures. Building on the richer contrast of optical‐diffraction tomography (ODT), Ryu et al. [17] trained an anchor‐free FCOS detector [85] on 1224 volumetric RI stacks (112^3^ voxels) containing more than 115k annotated cells from 171 blood samples. The network, ResNet‐18 for localization followed by a small 3D CNN for typing, reaches 97% accuracy and 0.97 weighted‐F1 on a 10% test split, while previewing each cell in about 150ms on a single NVIDIA TITAN GPU. Its volumetric input lets it recover dry mass and morphological cues simultaneously and, by dispensing with anchor boxes, handle objects that range from platelets to swollen erythrocytes without manual size heuristics. That flexibility is tempered by class imbalance (only 124 leukocytes in the entire set) and a broad “abnormal RBC” label that bundles several pathologies, making performance on rare cell types hard to gauge; moreover, the Tomocube HT‐2 hardware that supplies the 3‐D data is not yet commonplace outside research labs.

Lin et al. [81] tackled hemoglobinopathies by coupling quantitative‐phase digital holographic microscopy with an instance‐segmentation backbone. Using a Mask R‐CNN [86] fine‐tuned on 2001 healthy and 4268 thalassemic RBC phase maps, the pipeline first delineates each cell (mean IoU≈0.95) and then labels it healthy or thalassemic, achieving 97.8% accuracy and an F1 of 0.98 on a held‐out test set. The phase images were recorded at 60×/0.80 NA (pixel pitch≈0.27 μm, 2048 × 2048px); preprocessing comprised background subtraction, phase unwrapping, and min‐max normalization, after which the frames were tiled into 512 × 512 crops and augmented by ±10° rotations, mirroring, and slight elastic warps to equalize class balance. Because Mask R‐CNN yields per‐cell masks, the authors extracted phase‐shift statistics and analyzed them with SHAP and canonical‐correlation analysis, showing that optical volume, mean phase‐shift, and its variance are the dominant cues and correlate strongly (*ρ* > 0.85) with 3D morphological descriptors obtained from ODT reconstructions. The study therefore pairs real‐time detection with a level of explainability, yet it still uses a single‐center dataset (29 patients) and a 0.2 s GPU inference per megapixel frame, which constraints future multi‐center validations and TensorRT‐style acceleration could address.

The adaptability of DL models extends beyond immediate diagnostics, offering potential in longitudinal studies. Drawing on imaging‐flow cytometry's capacity for high‐throughput, label‐free capture, Doan et al. [43] assembled a longitudinal archive of millions of bright‐ and dark‐field single‐RBC images taken at 11–13 time points across the 42‐day storage life of 10 blood‐bank units. A ResNet‐50, fine‐tuned on only ~1% of that pool, classifies cells into the six canonical morphology states that trace storage lesions; the remaining 99% of images serve as an unbiased timeline on which the network's predictions are projected with t‐SNE and diffusion‐map embeddings to visualize the disc‐to‐sphere drift. By replacing manual gating with automated inference, the pipeline condenses what had been hours of cytometry review into minutes, and the continuous embeddings reveal subtle, donor‐specific aging trajectories that discrete class labels would obscure. Those advantages come with trade‐offs: minority crenated subclasses still accumulate mis‐labels, a single‐center cohort raises questions of generalizability, and widespread adoption hinges on access to imaging‐flow cytometers and the expertise to curate a starter set of ground‐truth masks.

Jiang et al. [82] coupled coded‐LED QPI with a two‐layer stacked sparse auto‐encoder to classify unstained RBC morphologies (Discocyte, echinocyte, spherocyte). After an adaptive Otsu + watershed segmentation step, the authors introduced a boundary‐curvature metric that flags irregular perimeters before classification. This edge descriptor trimmed split/merge errors and improved the model accuracy to 97.3% for 13 400 cells while processing a 1.9‐Mpixel phase frame in roughly 5 s. The study demonstrates that lightweight, non‐convolutional networks can rival CNN baselines in label‐free QPI when paired with task‐specific geometric features, although the limited variability in the dataset due to single‐center and residual confusion among highly pleomorphic echinocytes warrants that broader validation is still needed (Table 3).

**TABLE 3:** Sample deep learning methods for segmentation and classification in unstained blood smear analysis. These works show the evolution toward automated, end‐to‐end feature extraction, segmentation, and classification. Unlike traditional ML approaches, DL models eliminate the need for manual feature engineering, which enables them to adapt better to diverse and complex datasets. However, DL methods still face challenges related to data scarcity, computational complexity, and interpretability.

Article	Method	Performance	Strengths	Challenges
Jiang et al. [82]	Classification of RBC subtypes (differential‐phase‐contrast QPI with a coded‐LED array microscope) from donors Watershed algorithm for cell segmentation Application of a stacked sparse autoencoder and a softmax classifier Introducing a novel edge‐based descriptor **Dataset:** $\left(50\times 50\right)$ Train: 10800 cropped cells, 3600 per class Test: 3600 cropped cells + 6 full field QPI frames $\left(1920\times 1080\right)$ Augmentation: rotations, flips, and Gaussian noise (≈32 000 images)	Overall accuracy: **97.3%** Mean misrecognition of six unseen full‐field QPI frames = **7%** Throughput: 25–30 cells per second	True label‐free workflow Boundary‐curvature descriptor cuts mis‐splits and reduces misclassification Runs fast on CPU‐only hardware	Echinocyte shape diversity drives most errors Small, single‐center dataset (< 4000 unique cells despite augmentation) Limited interpretability compared with CNN/Grad‐CAM style models
Huang et al. [87]	Classification of activated and inactivated neutrophils obtained through spiral microfluidic WBC sorter through oil‐immersion bright‐field microscope Thresholding through Otsu WBC classification using pre‐trained ResNet50 **Dataset:** $\left(200\times 200\right)$ 25 396 single‐cell crops, 8621 granulocytes, 8384 lymphocytes, 8391 monocytes Augmentation: rotation and flipping ≈10 000 images per class 70–15‐15 train/val/test split	Test metrics: Accuracy = **94%** Precision = **96%** Recall = **90%** F1 = **93%** Granulocyte identification (activated vs. inactivated): Aspect Ratio Metric: > 1.2 for activation. Roundness Metric: > 0.76 for confirmation of activation state.	High‐resolution images Micro‐fluidic spiral channel yields clean, label‐free separations Novel approach of combining aspect ratio and roundness metrics offers quantitative analysis of neutrophil activation	All granulocytes are treated as neutrophils (basophils/eosinophils ignored) Activation state still relies on handcrafted aspect‐ratio and roundness thresholds
Chen et al. [84]	Classify WBC obtained through spinal microfluidic separation with MDDS double‐spiral chip in bright‐field microscope Use custom CNN that fuses low‐level (texture/contour) and high‐level semantic features Otsu segmentation to crop single cells and intensity normalization **Dataset:** $\left(200\times 200\right)$ 2195 cells, 1540 granulocytes, 444 lymphocytes, 211 monocytes Augmentation: rotations + mirrors; 31 058 images (≈10 300 per class)	Test metrics: Accuracy: **80.3%** Precision: **81%** Macro‐F1: **80%**	Double‐spiral micro‐fluidic chip enriches WBCs without stains Feature‐fusion retains nuclear detail through Grad‐CAMs (clear contours) Class imbalance is addressed by augmentation	Accuracy lags stained‐smear baselines (> 90%) Subtle inter‐class morphology (e.g., mono‐vs‐lymph) remains hard Heavy rotation/flip augmentation may inflate test scores
Ryu et al. [88]	Use optical diffraction tomography (ODT), RI tomograms at 0.162 μm/0.731 μm (xy/z) resolution to classify WBC Employ a modified 3D CNN model Comparative benchmarking between ML and 2D CNN models **Dataset: (171 blood samples, 1224 tomograms)** 115 143 cell instances, 96 860 RBC, 9142 platelet, 9017 abnormal‐RBC, 124 WBC 70–20‐10 train/val/test split External test set; 1539 tomograms for CNC‐parameter validation	Detection mAP: **0.977** Weighted‐F1: **0.9708** Total accuracy: **0.9712** Inference: 150 milliseconds per cell	True 3D RI tomograms, offering simultaneous morphology and biochemical indices Smallest backbone (ResNet‐18) already tops accuracy with 21 M params	Severe WBC data imbalance (124 vs. 115 k cells) drags precision/recall “Abnormal RBC” vs. “RBC” decision boundary subjective Requires ODT hardware not common in clinics
Doan et al. [43]	Label‐free classification of RBC morphologies obtained Amnis ImageStream X using bright and dark‐field channels Use a ResNet50 to classify RBC **Dataset:** $\left(50\times 50\right)$ 10 red‐cell concentrate units, 11–13 sampling days over 42‐day storage > 100 000 cells per aliquot, over 3.4 M paired images in total 1.7% of pooled A/B/C/D/E/F/H bags used to train ResNet50 Iterative bag‐wise cross‐validation	Accuracy: **89.4%** Macro‐recall**: 88%–92%** for most classes, **80%** for crenated‐discoid Morphology Classification: Smooth Disc, Crenated Disc, Crenated Sphere: > 90% recall Crenated Discoid, Crenated Spheroid: 80%–86% recall	First DL model targeting storage‐lesion continuum IFC platform gives high throughput and multi‐channel context Augmentation methods for rare classes (smooth discs) improve classification performance	Low recall for crenated‐families Manual gating still needed for feature selection Single blood‐bank cohort limits external validity Model trained on 10‐bag split from one facility

By stacking wavelength‐specific optics with data‐balancing tricks, Lebel et al. [89] show that label‐free images carry enough intrinsic contrast for stage‐level malaria diagnostics: deep‐UV maximizes resolution, while near‐UV (405 nm) provides hemoglobin‐driven absorption that lights up ring forms even when phase contrast cancels at other planes. The same design, however, ties performance to hardware: the best numbers rely on a quartz flow cell, sub‐300‐nm illumination and an anchor‐free FCOS detector running on an NVIDIA V100, hardware that is still scarce in routine labs. Class imbalance also persists. Only 124 white‐cells appear in the entire 3D ODT set, so precision/recall for leukocytes is unreported, and the “abnormal RBC” bucket collapses multiple pathologies into one. Nonetheless, by open‐sourcing code and showing that a €200 LED upgrade to a standard microscope reproduces sub‐0.1% limits of detection, the study removes the last procedural barrier, staining, from high‐throughput malaria counts and makes a compelling case for moving label‐free diagnostics out of the optics lab and into the clinic.

Transfer learning is a technique in DL where a model pre‐trained on a large dataset is fine‐tuned for a specific task with a smaller dataset. Instead of training a model from scratch, transfer learning allows knowledge gained from solving one problem to be repurposed for another. This process involves using a pre‐trained network as a feature extractor, where the initial layers are often frozen, meaning their weights do not change during training. This prevents the loss of generalizable features while allowing the later, more specialized layers to be fine‐tuned for the new task. Additionally, the final layer is usually replaced to align with the new dataset's objectives.

Transfer learning has proven highly effective in blood cell analysis [90, 91]. For instance, Huang et al. [87] show that a modest, label‐free pipeline built on transfer‐learning ResNet‐50 can classify white‐blood‐cell subtypes and flag neutrophil activation in real time. WBCs are first enriched with a single‐pass spiral micro‐fluidic chip, imaged in bright‐field at 100×, segmented by Otsu, then rotated and mirrored to yield ≈25k single‐cell crops (balanced across granulocytes, lymphocytes, monocytes). Fine‐tuning only the last residual block lifts performance to ~94% accuracy (F1 ≈ 0.93), and deeper backbones (ResNet‐101, Inception‐V3) give no further gain; inference stays below 100 ms per cell. Among the granulocyte outputs, a simple aspect ratio > 1.2 and roundness < 0.76 rule marks elongated neutrophils as “activated,” enabling stain‐free immune monitoring minutes after draw. The method runs on stock optics and avoids hand‐crafted features; however, it still folds eosinophils into the granulocyte class and depends on tight spiral‐chip tolerances for reproducible WBC isolation.

Despite significant advancements in DL, several challenges remain in the field of unstained blood smear analysis. These include data imbalance, the limited availability of annotated datasets, and high computational demands. A critical issue is the scarcity of publicly available datasets, which hampers the development and validation of robust models. Additionally, DL models often struggle with generalizability, as performance can vary significantly across different datasets and imaging conditions. Integrating DL into clinical practice necessitates addressing these challenges, with a focus on improving interpretability, computational efficiency, and adaptability. Ensuring transparency and explainability is essential for building trust among medical professionals and promoting the adoption of these models in real‐world diagnostic workflows.

## 7 Conclusion

Unstained blood smear analysis presents a unique set of challenges and opportunities in clinical diagnostics, requiring robust analytical methods to accurately identify cellular structures without the aid of chemical staining. Compared to traditional stained methods, unstained imaging preserves native cellular morphology and avoids staining‐related artifacts that could obscure critical features. This advantage not only eliminates the need for time‐consuming and costly staining procedures but also reduces dependency on expert pathologist annotations, potentially making the diagnostic process faster and more efficient, particularly in resource‐limited settings. Additionally, by leveraging intrinsic cellular properties such as refractive index and texture, unstained techniques provide high‐resolution images that capture subtle morphological and biophysical differences, offering valuable insights into cellular health and disease states.

However, despite these benefits, unstained imaging still presents significant challenges. The absence of contrast‐enhancing stains can make it difficult to differentiate between cell types, especially in heterogeneous or overlapping samples. This limitation necessitates the development of advanced image processing algorithms and robust ML and DL models capable of extracting meaningful features from low‐contrast images. Enhanced contrast enhancement methods and precise segmentation techniques are critical for addressing issues such as low transparency and the complexity of assessing overlapping cells.

Traditional rule‐based methods continue to play a role in blood smear analysis due to their simplicity, computational efficiency, and interpretability. These methods excel in preliminary analyses and scenarios where rapid processing is crucial, offering a reliable option in settings with limited computational resources. Nevertheless, their performance can degrade in complex environments involving noisy images, overlapping cells, and heterogeneous morphologies. Despite these limitations, rule‐based approaches maintain value due to their deterministic nature and ease of implementation, particularly where transparency in decision‐making is essential.

ML techniques have bridged some of the limitations of rule‐based methods by leveraging data‐driven feature extraction to handle more complex diagnostic scenarios. ML models demonstrate improved robustness and adaptability by utilizing handcrafted features such as cell shape, texture, and intensity. While these models offer increased flexibility, their reliance on domain expertise for feature engineering and limited generalizability across diverse datasets remain challenges. Moreover, ML methods still struggle with highly overlapping cells and require consistent imaging conditions to maintain performance. Unlike DL models, which continue to improve as more data becomes available, traditional ML models often reach a performance plateau even with additional data, as their effectiveness is bounded by the quality and representativeness of the manually engineered features.

The advent of DL has significantly transformed blood smear analysis by enabling automatic feature extraction and end‐to‐end learning directly from raw image data. CNNs and transformer‐based models have demonstrated state‐of‐the‐art performance in segmentation and classification tasks, consistently outperforming traditional ML models. Unlike ML approaches, DL models autonomously learn hierarchical and abstract feature representations through multiple network layers, capturing intricate patterns that might be overlooked by manual feature engineering. However, DL models also face persistent challenges, including the need for large, annotated datasets, particularly difficult to obtain in medical imaging. Additionally, their high computational costs and “black‐box” nature raise concerns about interpretability and transparency, which are critical for clinical acceptance. Ensuring generalizability across diverse imaging systems and clinical conditions remains a crucial research focus.

A further obstacle is the fragmented landscape of datasets and evaluation protocols. Most label‐free studies rely on small, institution‐specific QPI, DHM, or ODT collections, and rapid innovation in optics shifts sensor formats and noise profiles faster than repositories can stabilize. This makes it unlikely that a single “ImageNet‐scale” archive will emerge soon; instead, modality‐aware sub‐benchmarks, rich metadata reporting, and distribution‐sensitive metrics (weighted‐F1, AUROC, calibration error) are needed to allow fair comparison and reproducibility across laboratories. Community efforts to publish even anonymized thumbnails and mask annotations would accelerate algorithmic progress and facilitate transfer‐learning between centers.

Looking forward, pairing these open benchmarks with explainable DL, lightweight inference hardware, and accreditation‐ready validation pipelines will be essential for translating biophotonics‐plus‐AI from proof‐of‐concept to routine hematology. As optical platforms continue to evolve, success will hinge on adaptable algorithms, transparent metrics, and shared data that together can bridge the gap between innovation and bedside practice.

## Author Contributions

Husnu Baris Baydargil was involved in conceptualization, investigation, writing the original draft, and editing. Thomas Bocklitz was involved in conceptualization, writing the original draft, project management, review, and editing.

## Conflicts of Interest

The authors declare no conflicts of interest.

## Data Availability

Data sharing is not applicable to this article as no new data were created or analyzed in this study.
